# Incorporating zinc ion into titanium surface promotes osteogenesis and osteointegration in implantation early phase

**DOI:** 10.1007/s10856-023-06751-1

**Published:** 2023-11-02

**Authors:** Xutengyue Tian, Peng Zhang, Juan Xu

**Affiliations:** 1 Department of Oral and Maxillofacial & Head and Neck Oncology, Beijing Stomatological Hospital, Capital Medical University, Beijing, China; 2 Shenzhen Institutes of Advanced Technology, Chinese Academy of Sciences, Shenzhen, China; 3 Shenzhen Engineering Research Center for Medical Bioactive Materials, Shenzhen, China; 4 University of Chinese Academy of Sciences, Beijing, China; 5 Department of Stomatology, Sijing Hospital of Songjiang District, Shanghai, China

**Keywords:** Zinc, Titanium, Surface modification, Biocompatibility, Plasma immersion ion implantation, Bone-implant ground section staining

## Abstract

The objective of this study is to further investigate the feasibility of Zinc–Titanium implant as a potential implantable material in oral application in aspects of osteoblast biocompatibility, osteogenesis and osseointegration ability. First, we used plasma immersion ion implantation and deposition (PIIID) technology to introduce Zinc ion into pure Titanium surface, then we used X-ray photoelectron spectroscopy to analyze the chemical composition of modified surface layer; next, we used in vitro studies including immunological fluorescence assay and western blotting to determine responses between MG-63 osteoblast-like cell and implant. In vivo studies adopted pig model to check the feasibility of Zn–Ti implant. Results showed that in vitro and in vivo were consistent, showing that Zn ion was successfully introduced into Ti surface by PIIID technique. The chemical and physical change on modified plant resulted in the more active expressions of mRNA and protein of Type I collagen in MG-63 cells compared with non-treated implant, and the better integration ability of bones with modified implant. We confirmed the Zn–Ti implant owns the ability in promoting osteogenesis and osteointegration in early phase of implantation and is a qualified candidate in dentistry. The overview of our study can be depicted as follows.

**Graphical Abstract:**

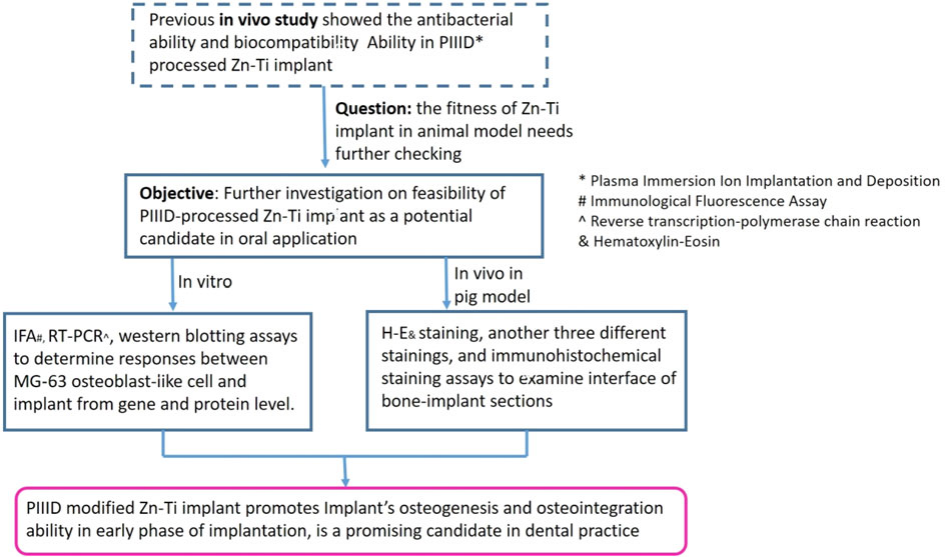

## Introduction

Dental implant is considered the mainstream strategy for oral restoration in recent years, as dental implant could simulate the function of natural tooth to maximum. The long term survival of dental implant is recognized as a successful dental implant, and such success is largely depending on implant primary stability (i.e. mechanical stability) and, the biological stability (i.e. osseointegration between bone and implant) [1]. Several factors affect implant primary stability, including bone density, implant design, and surgical technique [2]. As to biological stability, inflammation and infection around the implants, leading to peri-implantitis and loss of supporting bone, may eventually lead to implant failure. Surface chemistry of the implant and lack of cleanliness on the part of the patient are related to peri-implantitis [3]. Therefore, the ideal orthopedic implant is expected to have good mechanical properties and good biocompatibility, to inhibit microbial infection and to promote osseointegration [4].

Commercially pure titanium (cp-Ti) and its major medical alloy dominate all kinds of implants due to its superior properties. Surface treatment and modification on titanium implants are needed to improve tissue bonding and inhibit bacterial adhesion [5]. These modification include biochemical methods (i.e.to immobilize anti-bacterial macromolecules, antimicrobial peptides or antibiotics onto implant interfaces via bio-film formation), physical and chemical methods (i.e. coating, grit blasting, acid etching, and sand blast, etc), each methods has its advantages and own limitations [6, 7]. Among physical methods, plasma treatment can significantly alter metal surfaces’ physicochemical properties, such as surface chemistry, roughness, wetability, surface charge. Commonly seen plasma treatment techniques includes plasma spraying, plasma immersion ion implantation and deposition(PIIID), plasma vapor deposition, and plasma electrolytic oxidation [8, 9]. Plasma Immersion Ion Implantation and Deposition (PIIID) technology enables to embed various element into the near-surface region of various substrate. By means of direct ion immersion techniques, PIIID has extra advantages in facilitating to manipulate complicated and delicate industrial components in batches in fast speed, moreover, it can strictly control the concentration and depth distribution of implanted ions in substrate by adjusting the implantation parameters [10–12].

Zinc is an essential trace element that is crucial for growth, development, and the maintenance of immune function [13]. Introduction of Zn ion (Zn^2+^) on titanium dental implant, i.e. Zinc-containing titanium coating has been reported to promote osteogenic activity and pro-angiogenic ability, to inhibit the inflammatory response [14–16]. Most distinguish, Zn^2+^, exhibits excellent anti-bacterial performance [17–19]. to the best of our knowledge, the intrinsic link under the effects of Zn^2+^ in titanium implants to improve new bone formation and osseointegration is not well understood. More investigations are necessary to assess its effectiveness and safety in humans and to establish a standard methodology and ideal compound for incorporating zinc ion into titanium implant surfaces in a clinical setting.

The objective of this study is to investigate the feasibility of Zinc-Titanium (Zn-Ti) implants as a potential dental implant material in oral application in aspect of anti-bacterial ability, osteoblast biocompatibility, osteogenesis and osseointegration ability via in vitro and in vivo means. In our previous studies [20–22], we already successfully adopted PIIID technique to immerse and deposit Zn^2+^ into native Ti implant. Physical analysis represented that Zn^2+^ immersion brought to the roughness and porous “honeycomb” structure on Ti surface and such modification showed anti-bacterial property by deterring bacterial adhesion. Consequently, in vitro assays were introduced on the interaction between MG-63 osteoblast-like cell and implant, results suggested the Zn-Ti implant could promote cell proliferation.

Based on these, here we use animal model as a follow-up to study Zn-Ti implant.

Firstly, we conducted in vitro studies to check MG‑63 osteoblast-like cells interaction with modified implant from mRNA and protein level. Further, we test implant biocompatibility in pig model, including histological observation of bone-implant sections and the immunohistochemical staining of Type I collagen on bone-implant slicing. Results were consistent with in vitro data. We confirmed that Zn-Ti implant promotes osteogenesis and osseointegration in early phase of implantation, and can be a compelling candidate in dentistry clinic practice.

## Materials and methods

This study, including all methods and experimental protocols, was performed in accordance with the hospital ethical guidelines and the ARRIVE guidelines, and approved by the Scientific Investigation Board of the General Hospital of the People’s Liberation Army, Beijing, China.

### Preparation of Zn-implanted titanium

Commercial class-4 pure titanium (Ti) rods (TA3 purity 99.99%, purchased from Baoji Nonferrous Metal Processing Factory, Baoji, Shanxi, China) were fabricated into disc-shaped samples in a diameter of 10 mm and thickness of 1 mm. They were then ground and polished by a grinding machine to an average surface roughness of 0.4 μm till mirror-like status, and then sonicated sequentially in acetone and anhydrous ethanol (20 min per each). These rods were parted into two groups, half of the disks (referred to as “cp-Ti” commercial pure control group) were without any Zn ion implantation and deposition treatment, and were stored in a sealed container for subsequent use. The other disks (referred to as “Zn-Ti” group) were subjected to Zn ion implantation in vacuum using a fourth-generation Plasma Immersion Ion Implantation and Deposition (PIIID) device developed by the State Key Laboratory of Advanced Welding Production Technology (Harbin Institute of Technology, Harbin, Heilongjiang Province, China). Each Zn-Ti disc was cleaned by argon ion sputtering for 10 min, followed by Zn implantation for 20-, 40-, 60- and 80 min. The implantation source included a pulsed cathodic arcplasma with the following parameters: the implantation pulse voltage (V) was 20 kV, the implantation pulse width (τ) was 300 μs, the Zn cathodic arc pulse width was 300 μs, and the operating pressure (P) was 0.1 Pa. All disks were sterilized with ethylene oxide before use.

### Surface chemistry characterization by X-ray photoelectron spectroscopy (XPS)

The chemical composition of Zn-Ti disc was characterized by XPS (VG ScientESCALab220i-XL, Thermo VG Scientific, Hastings, UK). XPS was performed with 300 W Al Kα radiation under 3 × 10^–9^ mbar vacuum (VG ScientESCALab220i-XL, Thermo VG Scientific, Hastings, UK). The excitation source was an Al Kα X-ray with approximately 300 W of power. The basic vacuum degree for the analysis was 3 × 10^−7^ Pa. The electron binding energy was calibrated against the C1s peak (284.8 eV) from trace hydrocarbon contamination.

### Cell culturing and inoculation

Human MG-63 osteoblast-like cells were purchased (Cancer Institute and Hospital, Chinese Academy of Medical Sciences, Beijing, China) and cultured. Zn-Ti disks and cp-Ti control disks, 12 pieces per each group per each experimental batch, were placed into a 24-well plate and then seeded with cell suspensions (2 × 10^4^ cells/ml) onto the surface. The disks were stand still for a short period, and 1 ml of α-minimum essential medium (α-MEM, pH 7.4, 100 ng/ml streptomycin, 0.4 M/100ml L-glutamine, 10% fetal calf serum, 100U/ml Ampicillin, 0.1 mM ascorbic acid) was slowly added to each well along the sidewall and the then incubated the plate in a condition at 37 °C with 100% relative humidity, and 5% of CO_2_ for 6-, 24- and 48 h each.

### The morphological observation of MG-63 cells by Scanning Electron Microscope

Regarding to the MG-63 cells, after culturing for 48 h, the Zn-Ti and cp-Ti disks were washed with 0.1 mol/L phosphate-buffered saline (PBS) and the cells were fixed overnight in 2.5% glutaraldehyde. Then, samples were dehydrated by a gradient ethanol (20, 50, 70, 90 and 100%) for 10 min each. Then, they were immersed in isoamyl acetate (a critical point drying fluid) for 1.5 min and finally sputter coated with a thin layer of gold/palladium. The morphology of attached MG-63 cells was visualized by Scanning Electron Microscope (SEM, Hitachi S‑520; Hitachi, Ltd., Tokyo, Japan).

### Analysis of the formation of type I collagen in MG-63 cells by Immunological Fluorescence Assay (IFA)

After incubation for 6, 24, and 48 h respectively, disks from each Zn-Ti and cp-Ti group were removed from the 24-well plate. Each disc was rinsed with 0.1 mol/L PBS for three times, fixed in 4% formaldehyde for 20 min, and immersed in 0.1% Triton X-100 for 20 min to increase cell permeability. After blocking nonspecific sites by immersion in 1% bovine serum albumin for 40 min, each specimen was sequentially incubated with rabbit monoclonal IgG, IgG recognizes human type I collagen (1:50 diluted at 4 °C in the dark overnight) and fluorescein isothiocyanate, FITC-labeled goat anti-rabbit IgG (1:200 diluted; 37 °C in the dark for 2 h). Each specimen was then mounted in an antifade medium to characterize the formation of Type I collagen under a laser confocal microscope. Twenty cells were randomly selected on each specimen to calculate the immunofluorescence pixel intensity (Leica Confocal software Version 2.61) image analysis (Leica confocal software 4.0, Ernst Benz Company Lai, Germany).

### Evaluation of type I collagen protein expression by Western Blotting assay

For quantitative analysis of Type I collagen protein expression, the total protein of MG-63 cells was extracted and protein concentration was determined according to BCA protein Assay Kit(Biomedical Technology Co.Ltd, Beijing, China) following the manufacturer’s protocol. Generally, certain amounts of protein from MG-63 cells were loaded onto a 10% SDS-PAGE gel, the gel was then transferred to a polyvinylidene difluoride membrane and incubated overnight at 4 °C with rabbit anti-type I collagen (1:50 diluted) and rabbit anti-β-actin antibodies (1:50 diluted). Membranes were then incubated with secondary antibody at room temperature for 2 h. The signals of Type I collagen protein expression was examined by an enhanced chemiluminescence detection system (Tanon Science & Technology Co., Ltd., Shanghai, China) according to the manufacturer’s instruction. To quantitative analyze the protein expression of MG-63 cells, following formula was used: Type I collagen protein expression = the band intensity of Type I collagen protein/the band intensity of β-actin.

### Preparation of disks with two sides of Zn-Ti and cp-Ti

The cp-Ti surface is processed by PIIID technique. For in vivo study, the disc implanting into animals was with two sides, designating as Zn-Ti side and cp-Ti side, respectively. The two sides are easy to discern by color since Zn-Ti side was pale yellow and cp-Ti was silver white. These samples were then sonicated and sequentially washed by acetone and anhydrous ethanol for 10 min each. Disks were stored in a sealed container, autoclaved for subsequent use.

### Establishment of large animal model

Healthy and matured, four-month old miniature pigs (gender ratio of 1:1 from Experimental Animal Research Center, China Agricultural University, Beijing, China), with weight of around 20 kg, were purchased in three different batches in an interval of approximately one month within three months. When pigs were raised to 5~7 months, we carried out surgery as follows: the miniature pigs were fasting for 12 h before surgery. They were injected with anesthetic of thiaminone at auricularis posterior in a ratio of 15 mg thiaminone per weight of 1 kg. The pig was laid in a lateral position, removing hairs, and the left and right mandibular lateral incisors were pulled out. The alveolar fossa incisions were trimmed to a diameter of 10 mm by high speed drill through tooth distal plane. Then, the disinfected disks were implanted into alveolar fossa at left and right sides and wounds were sutured up by number 4 surgical thread. In the following one week after operating the surgery, pigs were received the penicillin (1.6 million unit) by intramuscular injection twice per day to prevent the post-surgical wound infection. These experimental pigs were nurtured together with the control groups (i.e. pigs without any surgical treatments), and finally killed at time points of 4-, 8- and 12-week post-surgery for subsequent in vivo experiments.

### Preparation of histological specimen

Pig’s lower jawbone, comprising the dental implants at both left and right sides, was cut to a volume of about 2 cm^3^ by bone saw and residual soft tissues were eliminated. The bone was then fixed by 4% paraformaldehyde for 48 h, followed by washing under tap water for 24 h so as to rinse off the formaldehyde residual. The specimen was dehydrated by a gradient of ethanol from 70% to 100% and bleached in chloroform and soaked in wetting agent for immersion and embedding. Next, the specimen was prepared as undecalcified bone specimen by saw microtome (Leica 2500E) cutting to the thickness of 5 μm and baked at 42 °C overnight in oven. The specimen underwent deplasticizating by 2-ethoxyethyl acetate for 20 min for three times and dehydrated by graded ethanol for subsequent histological Hematoxylin-Eosin (HE) staining.

### Hematoxylin-Eosin (HE) staining

Specimen was dyed by hematoxylin for 6 min followed by water washing, 1% hydrochloric acid immersing for 3 seconds, water washing, immersing in 1% aqueous ammonia bluing buffer for 5 min, water washing, immersing in 0.5% eosin staining solution for 3 min, water washing, dehydrating through 90% and 100% ethanol, bleaching by dimethylbenzene, and embedded in neutral balsam for future microscope observation.

### The immunohistochemical staining of Type I collagen on bone-implant specimen

The prepared histological specimen was fixed in 10% paraformaldehyde solution for 48 h and then were decalcified in Ethylene Diamine Tetraacetic Acid (EDTA) solution for 2 months. After that, specimen was processed with paraffin embedding followed by cutting to section with thickness of 8 μm. The section was subject to the standard immunohistochemical staining of Type I collagen. Sections were de-paraffinized with xylene and rehydrated in alcohol and then incubated with 0.01 M citrate sodium for 20 min to eliminate endogenous peroxidase activity. Then, sections were incubated with rabbit monoclonal IgG, rabbit lgG recognizing human type I collagen (Neumarker, 1:50 diluted) at 4 °C overnight in darkness. Then incubated with HRP coupled anti-rabbit IgG (ab6721, Abcam, Shanghai, China)(1:200 diluted) at 37 °C in the dark for 60 min. Sections underwent diaminobenzine staining (DAB, Sigma) and bone cell nucleus was stained by hematoxylin solution.

### The preparation of bone-implant ground sections with three stain methods

The bone-implant undecalcified ground section was manufactured in mainly three steps. Firstly, sections with implant were cut to the thickness of 200 μm–300 μm by histotome, followed by three different staining methods (i.e. methylene blue-basic magenta, toluidine blue and Masson-Goldner), and last, sections were sliced to the thickness of 30 μm~50 μm by manual operation. Specific procedures were done referring to Wang’s [23, 24]. Microscope analysis then been performed on the ground sections to observe the newly regenerated bones adjacent to implant-bone interface. All digital images obtained from the stained sections were randomly chosen and analyzed.

### Statistical analysis

Data were reported as mean ± standard deviation (SD). Differences among groups were analyzed using SPSS16.0 statistical software (SSPS, Inc., Chicago, IL, USA). One-way analyses of variance followed by a Tukey test were employed to determine the statistical significance, which was accepted when *P < 0.05.

## Results

### Characterization of chemical composition of Zn-Ti disc surface

**Fig. 1:**
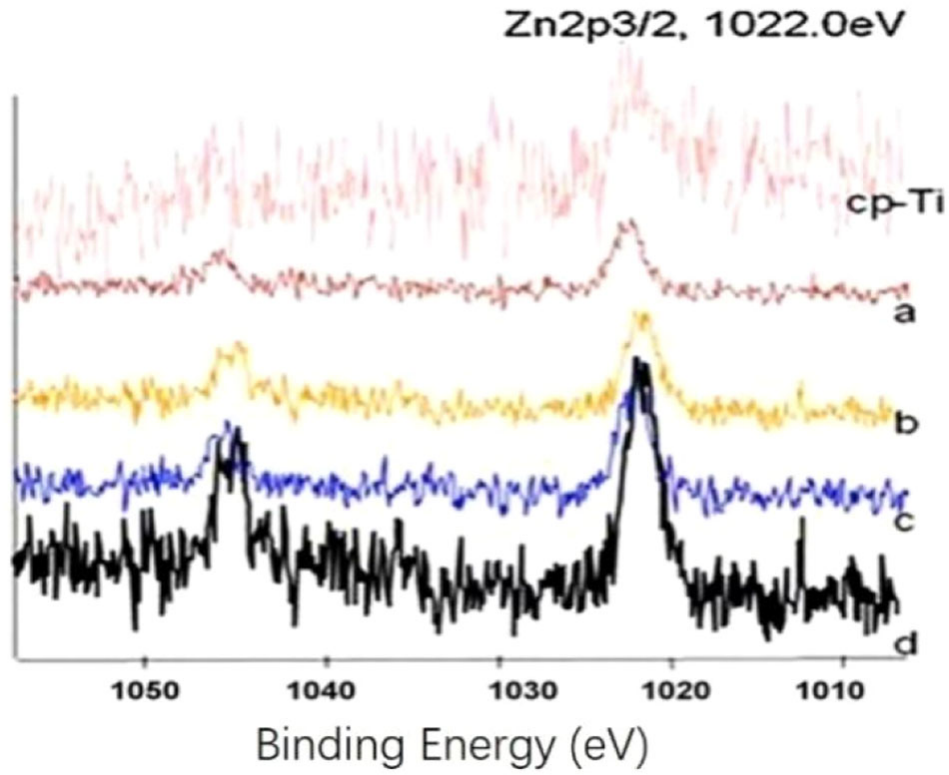
X-ray photoelectron spectra of the cp-Ti disc (control group), and the Zn-Ti disc surfaces. **a**–**d** represent 20, 40, 60 and 80 min Zn ion implantation-deposition time, respectively

As depicted in Fig. 1, Titanium (Ti), Oxygen (O) and Carbon (C) are the dominant signals on pure Ti surface (cp-Ti) while after the implantation of zinc ion, an overt signal of Zn appeared at the 1022 eV. In addition, along with the extending deposition time from 20 to 80 min (corresponding to Fig. 1 from d, c, b, a), the Zn signal intensity gradually increased and the maximum peak was detected at 80 min (Fig. 1a), and such increase weakened the signal Ti, O and C. Therefore, Zn-Ti-80 min disc was selected for the subsequent experiments. As to the Zn2p3/2 spectrum peak exhibited at cp-Ti disc surface, our explanation is this may due to the impurity substance of Ti materials [20].

### Alteration of MG-63 cell morphology on Zn-Ti disc surfaces

**Fig. 2:**
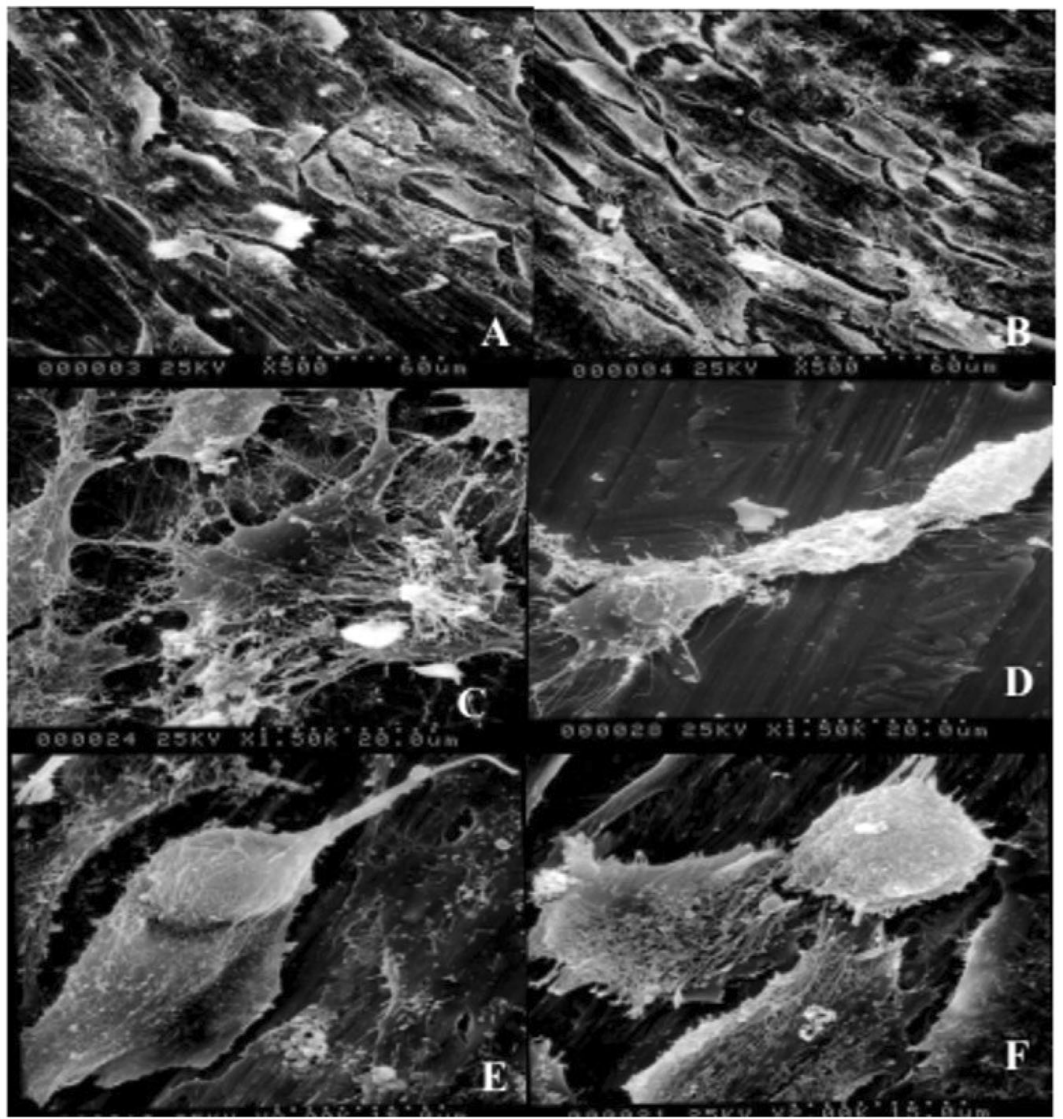
Scanning electron microscope (SEM) images of 48 h cultured MG-63 cells morphology on unmodified (cp-Ti) and Zn-modified Ti surfaces (Zn-Ti). **A**, **B** MG‑63 cells on cp-Ti and Zn-Ti disks with magnification of x500, respectively. **C**, **D** MG-63 cells on cp-Ti and Zn-Ti disks with magnification of x1500, respectively. **E** Secreted extracellular matrix (ECM) was visible covering the substrate granules (Magnification, x2000) and **F** cells undergo division were observed (Magnification, x2000)

The morphology of 48 h cultured MG-63 cells on both implants’ surfaces are examined under SEM, and cell morphological alterations are significant. As shown in Fig. 2A, C, majority of MG-63 cells on cp-Ti disks are observed as round and irregular polygon shapes with fewer and shorter fibrillar extensions, and with the ratio of the macro axis to the minor axis is usually greater than those of other cells growing in the culture flask. While the morphology of MG-63 cells on Zn-Ti surface is changed. These cells are well developed, better flattened and fully stretched, and they are tightly anchored on Zn-Ti disc surface by growing more fibrillary protrudes. By an increased cell to substrate contact ratio, the forming of these thin and long fibrillary pseudopodia can develop extensive networks of cytoplasmic processes with neighboring cells (Fig. 2B, D). Moreover, the extension of Zn ion deposition time resulted in a greater density of MG-63 cells on implant surface. These attached cells secrets considerable quantities of extracellular matrix (ECM) protein that covered the granular surface (Fig. 2E) and appeared to be more active in cell proliferation and differentiation (Fig. 2F).

### The formation of type I Collagen and the mRNA expression of type I collagen in MG-63 cells

**Fig. 3:**
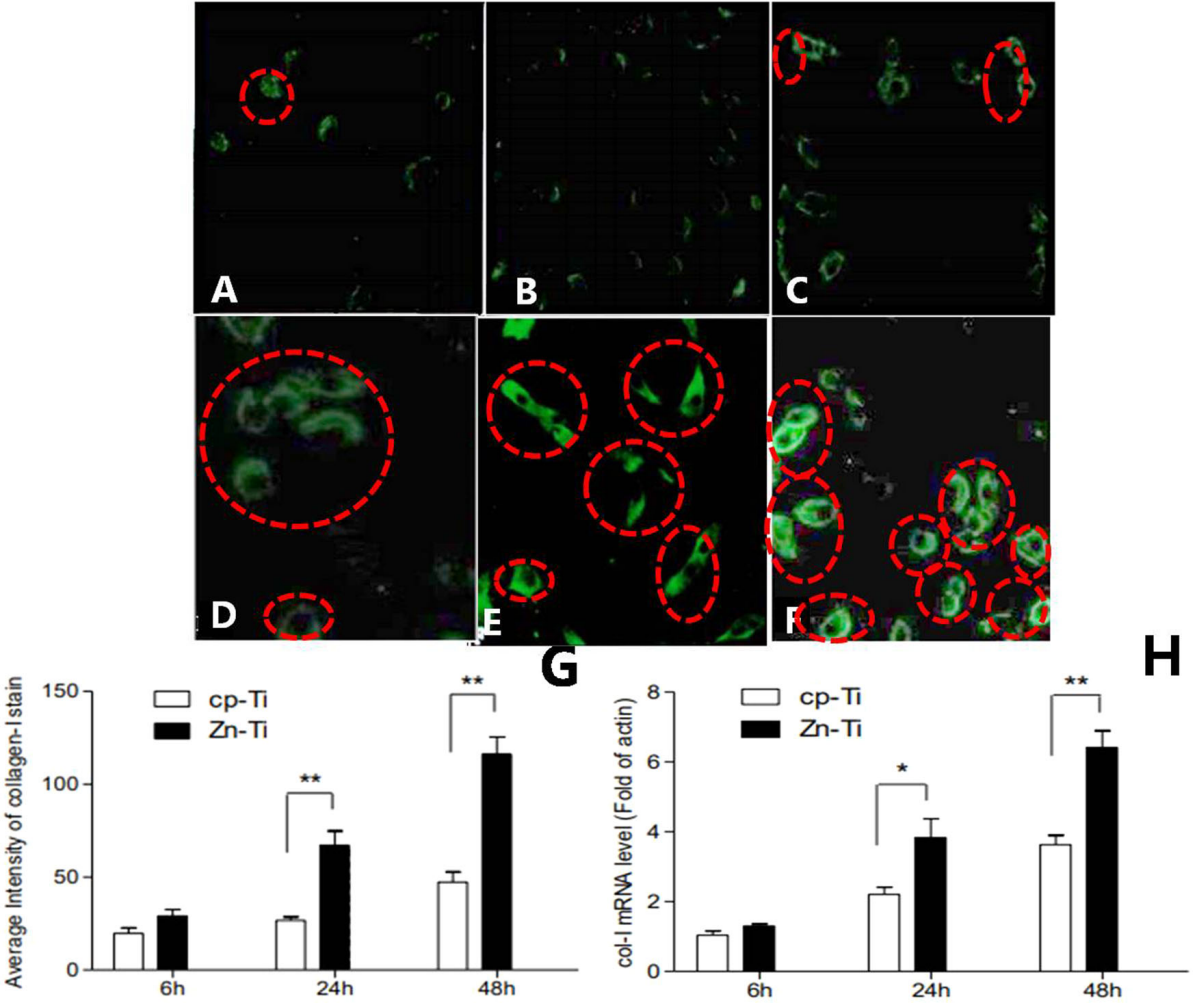
Fluorescence micrographs (Original magnification × 400) stained by fluorescence staining showing Type I collagen formed by MG-63 cells after a culturing time of 6, 24, and 48 h on cp-Ti disks, respectively (**A**–**C**), on Zn-Ti disks after a culturing time of 6, 24, and 48 h, respectively (**D**–**F**). Stained Type I collagen in cells were circled by dashed red line (**A**–**F**). Immunofluorescence pixel intensity of Type I collagen from MG-63 cells on cp-Ti and Zn-Ti surfaces. **P < 0.01, compared with cp-Ti (**G**). Quantitative measurement of Type I collagen mRNA expression from MG-63 cells on cp-Ti and Zn-Ti surfaces (**H**). *P < 0.05 and **P < 0.01, compared with cp-Ti

We use Immunological Fluorescence Assay (IFA) to analyze the formation of type I collagen in MG-63 cells. We selected Type I collagen as the indicator to evaluate osseointegration and bone formation in dental implant due to its vital role in osteogenesis. In Fig. 3, as shown in circled red line, both Zn-Ti and control cp-Ti disks displayed the existence of Type I collagens as they are stained green in MG-63 cells. The fluorescence intensity increases along with the increasing cell incubation time. The fluorescence staining is merely weak and can hardly been observed by eye on cp-Ti disc surface after 6-, 24- and 48 h MG-63 cell inoculation time (Fig. 3A–C), while that on Zn-Ti disc can be seen, though the intensity is also weak at 6 h, and more and more Type I collagen was fluorescence stained on Zn-Ti disks after 24- and 48 h cell inoculation (Fig. 3D–F). In quantitative study, after incubation for 24 and 48 h, the average Immunofluorescence intensity of Zn-Ti disc showed statistically difference to that of cp-Ti group (*P* < 0.01) (Fig. 3G). Figure 3H exhibited that both Zn-Ti and cp-Ti disks showed increased mRNA expression level along with longer MG-63 cell incubation time (from 6 to 48 h), While there was no statistic difference in both group after 6 h inoculation, and the mRNA expression level statistically differs after 24- and 48 h incubation time (*p* < 0.05 and *p* < 0.01, respectively). Noticeably, the mRNA expression level almost reached two-fold in 48 h in Zn-Ti group than that in cp-Ti group. Therefore, Zn deposited into Ti surface may induce osteogenesis from the early stage and further promote the osteogenic process.

### The protein expression of type I collagen in MG-63 cells

**Fig. 4:**
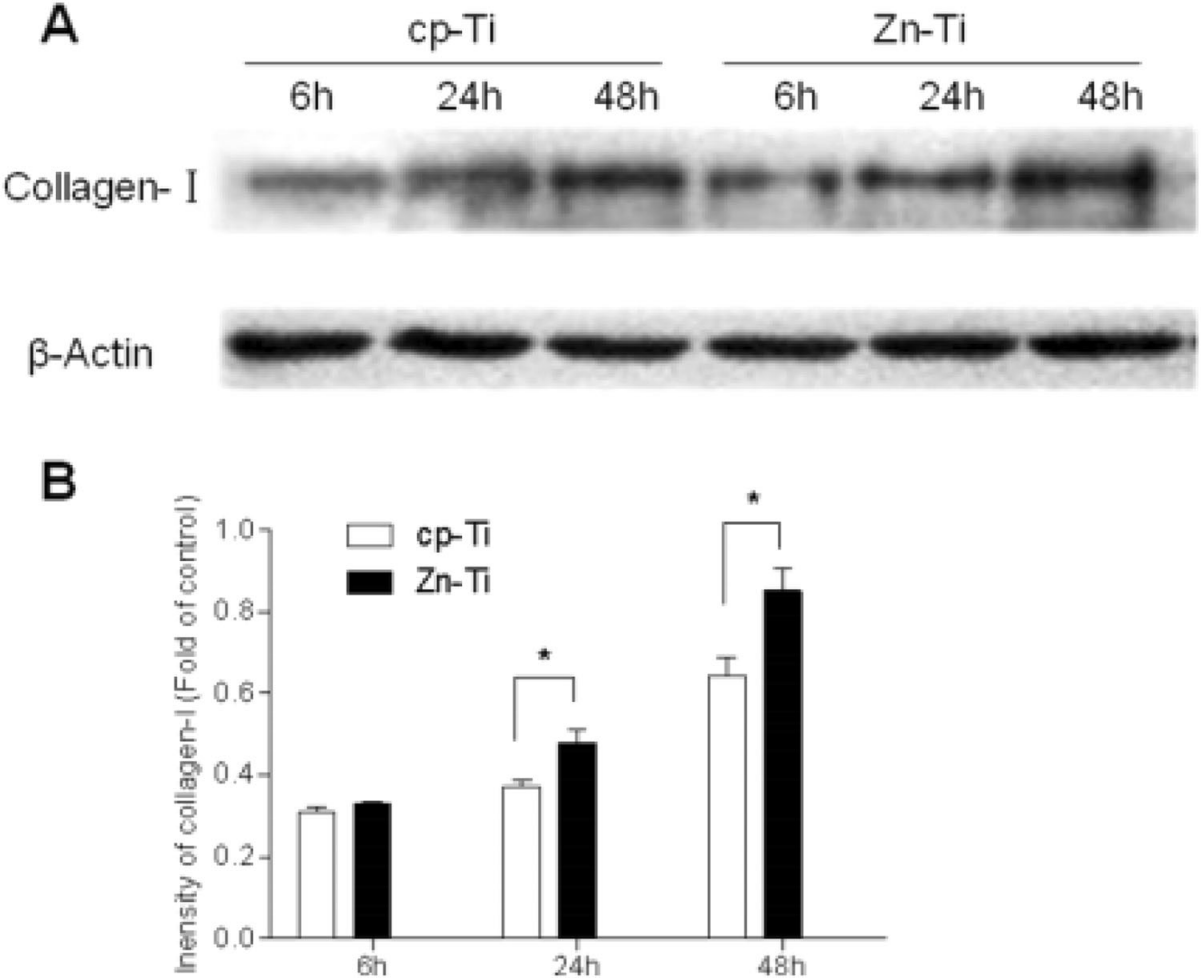
**A** Type I collagen protein expression in Zn-Ti and cp-Ti groups by Western blotting analysis. **B** Quantitive intensity analysis of Type I collagen protein from MG-63 cells on cp-Ti and Zn-Ti surfaces. *P < 0.05, compared with cp-Ti

The statistically higher expression of Type I collagen protein on Zn-Ti disks was confirmed by western blotting assay. Similarly, as that in mRNA expression pattern, along with longer cell inoculation time (i.e. 24- and 48 h), Zn-Ti disks revealed larger amount of protein expression and it was statistically significant compared to that of cp-Ti disc (Fig. 4).

### Histological and SEM observation of Zn-Ti implant in pig

**Fig. 5:**
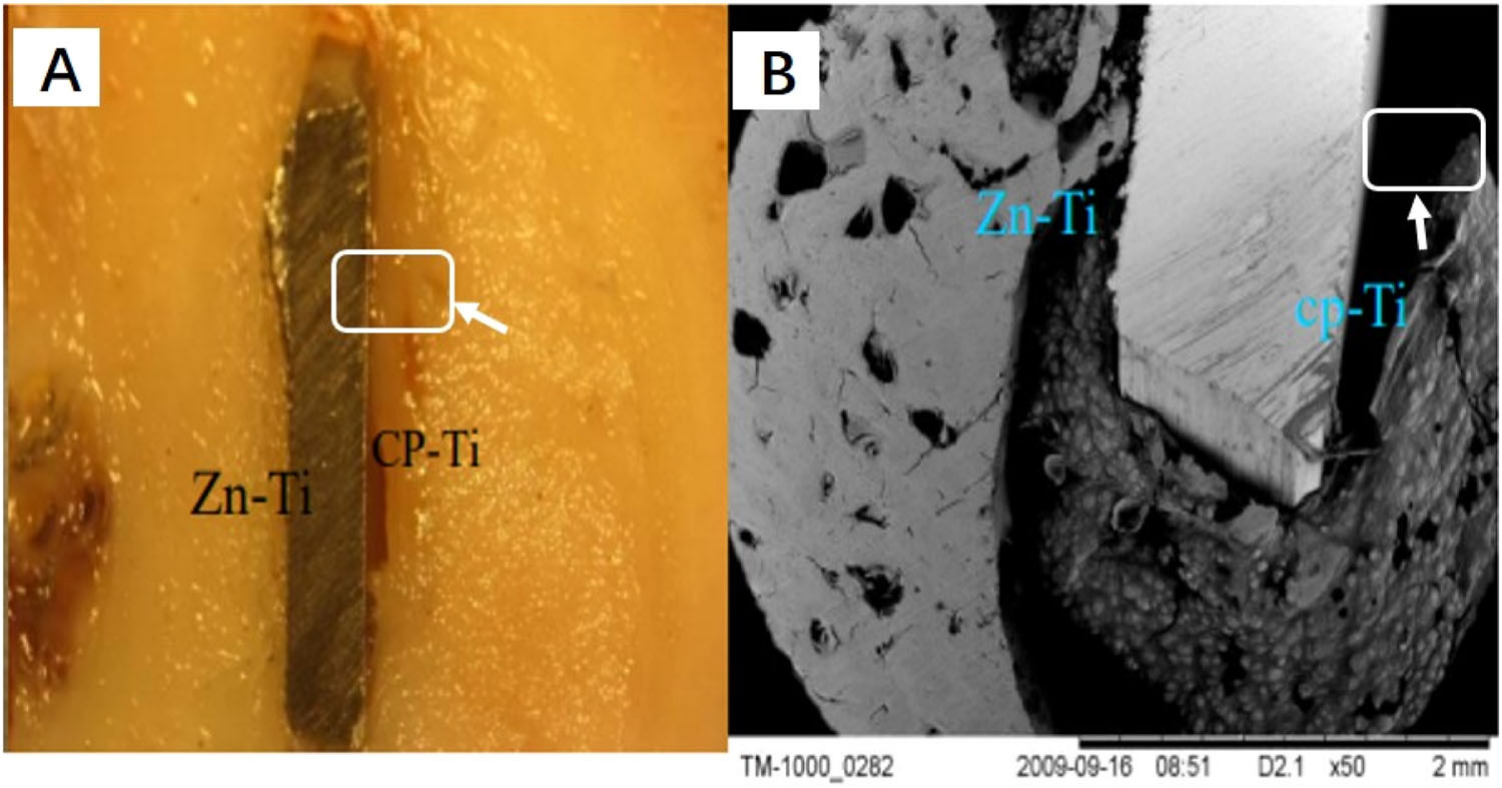
Four-week bone-implant disc with respective Zn-Ti and cp-Ti sides, observed by naked eye (**A**) and under SEM (X500) (**B**). A region of granulation tissue and fibrous connective tissue was blocked as an example, indicated by arrow

As observed by naked eyes in Fig. 5A, after 4-week implantation, the interface between Zn-Ti implant and newly regenerated bones is tightly combined, nearly no hyperplasia of inflammatory granulation tissue or fibrous connective tissue can be found. In contrast, there exists overt gap between the interface of cp-Ti implant and newly formed bones, and granulation tissue and fibrous connective tissues are distinct. The view under SEM (X500) shows regenerated newly bones grown at Zn-Ti interface in contrast with apparent gap at cp-Ti side (Fig. 5B). No such obvious difference was perceived in 8- and 12-week pig samples.

### HE staining results of histological specimen

**Fig. 6:**
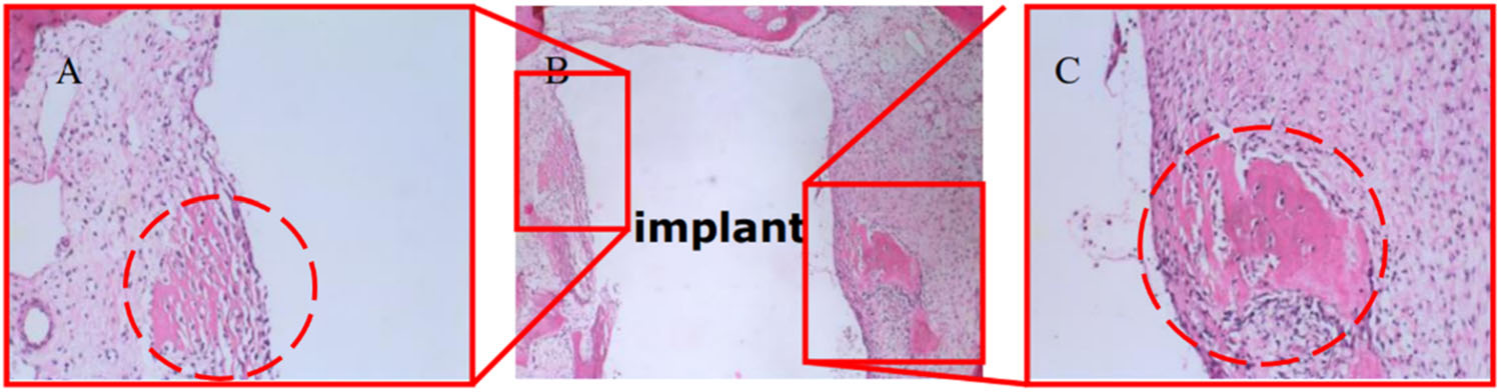
Microscopic views of 4-week implantation with Zn-Ti and cp-Ti sides after HE staining. **A** amplified view (X200) of Zn-Ti side. **B** full view (X50) of implant’s two sides. **C** amplified view (X200) of cp-Ti side. Regions of osteoblasts and osteoclasts were circled by red dash line in (**A**) and (**C**), respectively

In Fig. 6, 4-week bone-implant sample underwent HE staining and was examined under the microscope, results showed that more osteoblasts are viewed at interface of Zn-Ti side (circled by dash line in Fig. 6A) and more osteoclasts are found at cp-Ti side (circled by dash line in Fig. 6C). No such difference was observed at 8- and 12-week pig samples.

### Immunohistochemical staining of type I collagen on bone-implant section

**Fig. 7:**
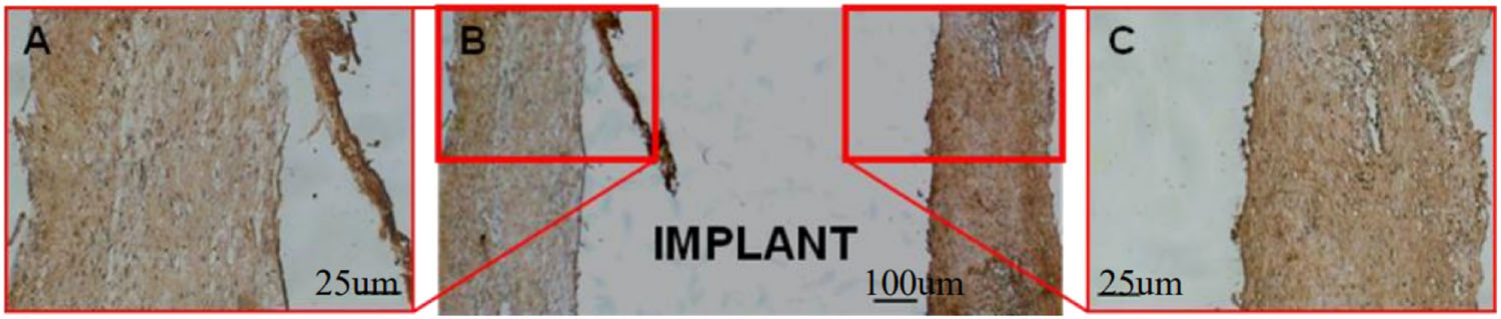
Immunohistochemical staining of Type I collagens after 4-week implantation. **B** the section view of bone-implant disc, interface of Zn-Ti is at the left and interface of cp-Ti is at the right. **A** Amplified view of Zn-Ti side. **C** Amplified view of cp-Ti side

According to immunohistochemical staining, Type I collagen and osteoblasts were intensively accumulated at Zn-Ti interface (Fig. 7A). They were rich in cytoplasm, indicating the active newly regenerated bone cells synthesis and secretion. By contrast, the present of these osteoblasts are rare at cp-Ti side (Fig. 7C). These in vivo results are in consistency with those of mRNA and protein expression in osteoblast-like MG-63 cells in in vitro study.

### Observation of hard tissue ground section after implantation

**Fig. 8:**
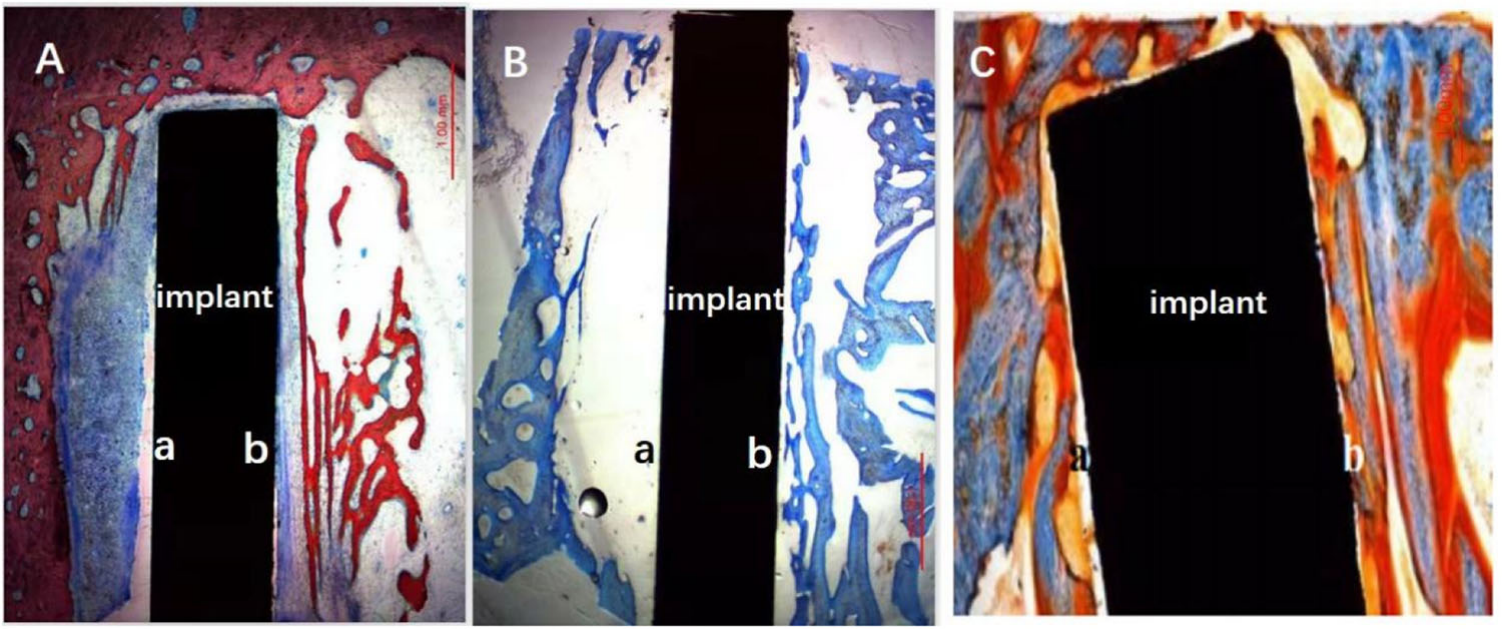
The staining results of bone ground sections with 4-week implants, processed by three staining methods. **A** sample stained by methylene blue-basic magenta solution. **B** sample stained by toluidine blue solution. **C** sample stained by Masson-Goldner solution. (a) Interface at cp-Ti and bone side in bone-implant ground sections. (b) interface at Zn-Ti and bone side in bone-implant ground sections

The implanted disks of 4, 8, 12-week were prepared as bone-implant ground sections separately and stained with different dying agents in order to evaluate bone tissue surrounding the implant. Distinctive tissue structures and features can be clearly observed and compared, representing histo-morphological condition of bone-implant interaction. In Fig. 8, in 4-week implantation section, three staining methods all showed the better integration ability of bones at Zn-Ti interface, and more newly formed bones appeared and narrower area between bone and Zn-Ti side. These differences were diminished along with the elongated implanting time. That was to say, there was no evidently differences in Zn-Ti and cp-Ti sides in 8- and 12-week samples bone-implant ground sections.

## Discussion

Surface modification is an economic and efficient way to adjust existing conventional biomaterials so as to meet the current and ever-evolving clinical needs. With the remarkable progress, the PIIID technology is widely used in biomaterial area in recent decades since it is with advantage in anti-bacterial property, and bacterial infections may lead to implant failure [25]. On the other hand, from an industrial perspective, the PIIID method is attractive owing to its capability of treating objects with irregular shapes, and with the various immersing ions elections. After PIIID processing of penetrating Zn ions into pure Ti material surface, it incorporates Zn ion into Ti surface, leading to the chemical composition and physical structure change comparing with non-treated Ti. Apparently, such physicochemical change of implant surface can largely influence the next step bone remodeling and finally affects the rate of osteogenesis and osteointegration in early phase after implantation [9–11]. Previously, we adopted PIIID technique to immerse and deposit Zinc ion into native Titanium surface, such modification brought anti-bacterial ability and better biocompatibility in in vitro study, but lacking of in vivo evidences. The highlight of this follow-up work lays in two aspects: first, in vitro experiments of interactions between human MG-63 osteoblast-like cells and modified implants were carried out, further confirmed previous results from mRNA and protein level; second, minipig was used as a large animal model for series of in vivo studies to check the feasibility of Zn-Ti implant. Therefore, our study was conducted in two parts. The first part was mainly the in vitro investigation of the interaction between Zn-Ti implant with cell on molecular level, the second part was the follow-up in vivo further confirmation.

In the first part of our study, we have detected the Zn ion composition after PIIID process and the Zn ion concentration increased along with the elongated implantation-deposition time. This has also shown in our previous study [20]. As to the physical structure change in modified Zn-Ti disc, we have observed the “honeycomb” structure on implant surface, the pores were formed due to the deposition of Zn ion and Zn ion penetration depth increased along with the deposition time [21]. By these analyses, we proved that the PIIID technique is able to incorporate Zn ion on Ti surface and bring chemical-physical change and such change is beneficial in oral implantation.

Cell adhesion and proliferation is important in dental implant since it directly and continuously influences subsequently cell growth, cell differentiation, and tissue regeneration. Many efforts have been put on characterizations of implant material biocompatibility in such aspect [26, 27]. In vitro studies to characterize the cell-material interactions are widely employed. In dental implantation, various studies already show that osteoblastis the dominant cell in osseointegration, and human osteoblastic line MG-63,which remaining stable in their phenotype over several passages in cell culture, is widely used when studying biocompatibility of implant materials [28]. In this study, we noticed the morphological difference of osteoblasts on pure Ti disc and Zn-Ti disc (Fig. 2). Therefore, we concluded that the better biocompatibility of Zn-Ti implant than that of pure Ti implant, however, such improvement is owing to the surface roughness or owing to the adoption of Zn ion remains unclear.

In the osteogenetic process, Type I collagen is secreted by osteoblastic cells, it is a key player in cell morphology and cell differentiation, and serves as a critical marker in osteogenesis. Type I collagen also functions as one of the constructive proteins in connective tissue and comprises mainly molecules of extracellular matrix, and is critical in cell anchor and adhesion [29]. Along with the formation of collagenous fibers, vesicles from osteoblastic cell cytoplasm comprising calcium and phosphorus will deposit on these fibers, so as to promote the bone mineralization by forming hydroxyapatite crystals [30]. In Kriegel A’s study, Type I collagen, serving asthe optimal carrier for bone sialoprotein, enhanced angiogenesis and osteogenesis by in vitro [31]. In other studies, increased roughness of implant surface helped to enlarge the cell-implant interface areas, which further increased the ion release ability and lead to higher mRNA gene expression and cell signaling in osteoblastic cells [32]. In our in vitro study, we have noticed the higher expression level of Type I collagen mRNA and protein in Zn-Ti implants (Figs. 3H, 4), which indicated the active interaction and integrating level between osteoblastic cells and Zn-Ti materials.

In the second part of our study, we implanted modified implants into mandibular lateral incisors of 5 to 7-month old miniature pigs, there are two reasons for such pig age selection: one is because the length of deciduous tooth root in miniature pigs is comparable to that of canine tooth root in humans’. The other is the tooth root resorption have not started in miniature pigs until 18-month old when tooth restoration happens. Thus, the bilateral mandibular lateral incisors in lower jaw of 5 to 7-month old miniature pigs is considered as an ideal implantation site.

In our animal model, SEM observation and histological studies of HE staining result showed that newly grown bone cells are closely united with modified side of dental implant (i.e. Zn-Ti side) with no overt gap, compared to the growth of granulation tissue and fibrous connective tissues at unmodified implant side (i.e. cp-Ti side), this indicated that the introduction of Zn ion by PIIID technique would not arise unwanted inflammatory reaction or hyperplasia of fibrous connective tissue (Figs. 5–8). The methylene blue-basic magenta, toluidine blue and Masson-Goldner staining methods are the dominate means to analyze the tissue structures of mature bone cells, regenerated new bone cells and osteoid, and the staining results can reflect the integration level of bone and implant in vivo condition, and whether dental implant is able to promote osteoblast cell growth. Since early dental implant failure was possible to occur in one month after implants inserting and most early dental implant failure was found by dentists by re-examinations, the implants should be removed when the early dental implant failure was found, this would not influence the later implantation [33]. We observed that after 4-week at the early implantation phase, osteoblast cells tended to migrate to the interfacial of Zn-Ti side, rather than the cp-Ti side, to integrate a direct bonding with implants, and these newly formed osteoblastic cells are abundant in cytoplasm, implying the very active bio-function of secretion and synthesis, no such observation was found at 8 or 12-week after implantation (Figs. 5–8).

In conclusion, via large animal model, our study further confirm that PIIID processed Zn-Ti implant could accelerate the osteogenesis, enhance interfacial biocompatibility and with shorter osseointegration time in early dental implanting phase. All of these results are consistent in vitro and in vivo to support the feasibility of Zn-modified Ti implant as a promising candidate in dental implantation.
